# The Top 12 Most Impactful Papers in Clinical Transplantation in 2025: TI Editors’ Choice

**DOI:** 10.3389/ti.2026.16247

**Published:** 2026-02-23

**Authors:** Thierry Berney, Chiara Becchetti, Maria Irene Bellini, Louise Benning, Ekaterine Berishvili, Oriol Bestard, Saskia Bos, Sophie Brouard, Federica Casiraghi, Antonio Citro, Lionel Couzi, Marta Jimenez-Blanco, Delphine Kervella, Wai H. Lim, Oriol Manuel, Nina Pilat, Stefan Schneeberger, Emilien Seizilles de Mazancourt, Nazia Selzner, Olivier Thaunat, Christian Toso, Arianna Trizzino, Dafna Yahav, Andrea Zajacova

**Affiliations:** 1 Editor-in-Chief, Transplant International; 2 Division of Transplantation, Immunology and Nephrology, Hospices Civils de Lyon, Lyon, France; 3 Ilia State University, Tbilisi, Georgia; 4 Associate Editor, Transplant International; 5 ASST Grande Ospedale Metropolitano Niguarda, Milan, Italy; 6 Deputy Editor-in-Chief, Transplant International; 7 La Sapienza Universita di Roma, Rome, Italy; 8 Assistant Editor, Transplant International; 9 Heidelberg University Hospital, Heidelberg, Germany; 10 University of Geneva Medical University Center, Geneva, Switzerland; 11 University Hospital Vall d’Hebron, Barcelona, Spain; 12 University Hospital Leuven, Leuven, Belgium; 13 INSERM U1064, University of Nantes, Nantes, France; 14 Executive Editor, Transplant International; 15 Istituto di Ricerche Farmacologiche Mario Negri IRCCS, Bergamo, Italy; 16 Diabetes Research Institute, IRCCS Ospedale San Raffaele, Milan, Italy; 17 Bordeaux University Hospital, Bordeaux, France; 18 University Hospital Ramon y Cajal, Madrid, Spain; 19 University of Western Australia, Perth, WA, Australia; 20 Centre Hospitalier Universitaire Vaudois, Lausanne, Switzerland; 21 Medical University of Vienna, Vienna, Austria; 22 Medical University of Innsbruck, Innsbruck, Austria; 23 Department of Urology, Saint-Louis Hospital, Paris, France; 24 Ajmera Transplant Center, Toronto General Hospital, Toronto, ON, Canada; 25 University of Geneva Hospitals, Geneva, Switzerland; 26 Azienda Ospedaliero-Universitaria Pisana, Pisa, Italy; 27 Sheba Medical Center, Ramat-Gan, Israel; 28 BREATHE, KU Leuven, Leuven, Belgium

**Keywords:** clinical transplantation, editors selection, game changers, impact, landmarks

The New Year is a perfect opportunity, not only for resolutions (that are unlikely to last very long), but also to reflect on the events of the past year and how they are likely to impact the future. In this piece, the editorial board of Transplant International is presenting what we think are the original articles in clinical transplantation published in 2025 that are the most likely to be game changers in our field in the near or not-so-near future. As the saying goes, “it is difficult to make predictions, especially about the future”, but despite the (very limited) risk, we are hopeful that we have not succumbed to hype in our selection, which was based on insights from members of the editorial board, but also on how well cited these papers already were. So, here are the top 12 papers, listed in Table 1, grouped by the topic they address. Selection is obviously a tricky task and for this reason, we will also mention other significant 2025 papers to provide the readers with a somewhat broader picture of what has happened in each of the selected topics (Supplementary Table S1).

**TABLE 1:** The top 12 most impactful papers in clinical transplantation in 2025.

Title	Topics	Journal	Corresponding author institution	Brief description
Transplantation of a genetically modified porcine heart into a live human [1]	Xenotransplantation Heart transplantation	Nature medicine	University of Maryland school of Medicine, Baltimore, MD, USA	Comprehensive report of the first pig-to-human heart xenotransplant using multi gene-edited porcine donors
Xenotransplantation of a porcine kidney for end-stage kidney disease [2]	Xenotransplantation Kidney transplantation	New England journal of medicine	Massachussets general Hospital, Boston, MA, USA	Comprehensive report of the first pig-to-human kidney xenotransplant using multi gene-edited porcine donors
Enzyme-converted O kidneys allow ABO-incompatible transplantation without hyperacute rejection in a human decedent model [3]	ABO incompatible tx Kidney transplantation	Nature biomedical Engineering	West China hospital, sichuan university, chengdu, China	Comprehensive report of the successful transplantation into a human brain-dead recipient of an A blood group kidney enzymatically converted into O blood group
Survival of transplanted allogeneic beta cells with No immunosuppression [4]	Immune tolerance Islet transplantation	New England journal of medicine	University of uppsala, uppsala, Sweden	Comprehensive report of the transplantation of gene-edited immune-evasive islet constructs into a type 1 diabetic patient
Stem cell-derived, fully differentiated islets for type 1 diabetes [5]	Stem cells Islet transplantation	New England journal of medicine	Vertex Pharmaceuticals, Boston, MA, USA	Report of a successful industry-led clinical trial of transplantation of embryonic stem cell-derived islet constructs into type 1 diabetic recipients
Cold perfusion vs. Static cold storage of deceased-donor kidneys — at 10 Years [6]	Machine perfusion Kidney transplantation	New England journal of medicine	University of groningen, groningen, The Netherlands	Ten-year data of a large randomized controlled trial of normothermic machine perfusion versus cold storage in kidney transplantation
Clinical outcomes with normothermic pulsatile organ perfusion in heart transplantation: A report from the OCS heart perfusion registry [7]	Machine perfusion Heart transplantation	Circulation	University of Utah, Salt Lake City, UT, USA	Registry data report on the feasibility and benefits of normothermic machine perfusion for DCD heart transplantation
Determining safe washout period for immune checkpoint inhibitors prior to liver transplantation: An international retrospective cohort study [8]	Transplant oncology Liver transplantation	Hepatology	University of Geneva Hospital, Geneva, Switzerland	Multicenter study providing safety data on the optimal cut-off for the wash-out period before liver transplantation after checkpoint inhibitor treatment
Insights from the BKEVER trial comparing everolimus versus mycophenolate mofetil for BK polyomavirus infection in kidney [9]	Infectious diseases Kidney transplantation	Kidney international	Strasbourg university hospital center, strasbourg, France	Randomized controlled trial exploring the impact of a switch from CNI to mTOR inhibitors in BK virus nephropathy
Donor-recipient mismatch at the SIRPA locus adversely affects kidney allograft outcomes [10]	Transplant immunology Kidney transplantation	Science translational medicine	University of Pittsburgh Medical school, piitsburgh, PA, USA	Exploration of the impact of SIRPA mismatch and of the innate immune system on kidney allograft rejection in two human cohorts and a mouse model
Continuous indices to assess the phenotypic spectrum of kidney transplant rejection [11]	Transplant pathology Kidney transplantation	Nature communications	Catholic university of Leuven, Leuven, Belgium	A banff paper providing strong evidence to replace threshold-based rejection categories with robust continuous indices
Extracorporeal photopheresis for the prevention of rejection after lung transplantation: a Prospective randomised controlled trial [12]	Photopheresis Lung transplantation	European respiratory journal	Medical university of Vienna, Vienna, Austria	Randomized controlled trial showing the benefit of adding extracorporeal photopheresis to standard immunosuppression for the prevention of CLAD

## Xenotransplantation

Xenotransplantation has repeatedly made the headlines in recent years since the near-simultaneous transplantation in the fall of 2021 of genetically modified porcine kidneys into human brain-dead recipients, or what is called the human decedent model [13]. The availability of genome-edited pigs has been the game changer in the field, allowing to significantly lower the immune barriers met in xenotransplantation [14]. The first clinical transplants using this resource were performed compassionately in 2022 in patients with no access to deceased donor organs and a compromised survival prognosis in the short term. Although the heart and the kidney xenotransplant recipients did not survive more than a few months, their causes of death not being fully clear, these first 2 cases provided evidence that the early phases of immune xenorejection had been harnessed [1, 2] thanks to the gene modification strategy, in particular the knocking off of 3 carbohydrate antigen genes known to cause hyperacute rejection (α 1-3 Gal, β 1-4 Gal, CMAH). The two featured articles provide a comprehensive narrative of the clinical course of the first heart recipient [1] and the first kidney recipient [2], from transplantation to death. While these cases cannot be considered successes from the patient perspective, they will provide invaluable information on the unforeseen hurdles met and how to overcome them [15]. In this regard, and despite the limitations of the decedent model [16], the in-depth analysis of the physiology, immunology and pathology of the first pig-to-human decedent kidney xenotransplant (single gene modification), using multi-omics analysis techniques [17, 18], and in particular the novel patterns of immune rejection unveiled and characterized, will also contribute to the better understanding of the physiological processes that will have to be tackled by the next-generation of protocols and gene-edited animals.

This paragraph would not be complete without mentioning the reports of gene-edited pig-to-human auxiliary liver transplantation in a living recipient, as a bridge to transplant [19], and lung transplantation using the decedent model [20]. Although the cell therapy procedure of islet transplantation would seem to have been an easier clinical model to explore in xenotransplantation, no attempt at transplanting genetically modified porcine islets has been reported to date [21].

## ABO-Incompatible Organ Transplantation

ABO-incompatible living-donor kidney transplantation has evolved into a standard option to expand the donor pool, but it usually requires desensitization to mitigate the risk posed by preformed anti-A/B antibodies. Protocols commonly include antibody removal (immunoadsorption/antibody adsorption and/or plasma exchange) alongside immunomodulatory therapy, adding complexity, cost, and potential morbidity despite outcomes approaching those of ABO-compatible living-donor transplantation. Kidney paired exchange is an alternative, although limited O-compatible availability may lead to extended waiting times for patients bearing the O blood group.

This paper reports the successful conversion of an A blood group kidney into O blood group, using *α-galactosidase* from *Bacteroides fragilis* during hypothermic machine perfusion. Three hours of machine perfusion were sufficient to remove >95% of blood group A antigens and allowed successful transplantation of the kidney in a decedent model without experiencing hyperacute rejection [3]. After A-antigen regeneration, antibody-mediated lesions and complement deposition were found starting 3 days post-transplant, but single-cell sequencing confirms the elevated expression of accommodation-related genes, suggesting the potential for longer-term tolerance [3]. This paper was published a few months after a previous similar report in the less common B-to-O combination, with similar outcomes [22]. These two papers provide compelling proof of concept that *ex vivo* antigen modification can safely expand the donor pool in ABO-incompatible living kidney donation and may therefore fundamentally reshape access in kidney transplantation. This strategy could also be employed in a deceased donor setting in the most urgent cases. While the validity of this method requires confirmation in the setting of clinical live donor kidney transplantation, it could also be explored in the more challenging model of liver transplantation, in which traditional techniques of pre-transplant ABO antibody clearance can be associated with deleterious outcomes [23].

## Immune Tolerance

The field of islet of Langerhans transplantation has seen 2 landmark clinical papers published last year. They represent a major step forward in beta-cell replacement therapies. The studies show that immune rejection and limited cell availability can be addressed. They set new benchmarks for safety, efficacy, and translational feasibility.

Importantly, they provide insights to guide the next-generation of durable islet replacement strategies. They also provide strategies that have the potential to be applied in the bioengineering of other organs.

The first is a proof-of-concept article in which the authors have applied their hypoimmune platform (HIP) approach to human primary islet cells. For the first time, they demonstrated the feasibility of gene-editing dissociated human islets using CRISPR-Cas12b and lentiviral transduction, to knock out HLA class I and II genes and overexpress the CD47 transmembrane molecule, which delivers a “*don’t eat me*” signal to cells of the innate immune system. These modified islet cells were then re-aggregated and transplanted into the forearm muscle of a patient with long-standing type 1 diabetes. In this first-in-human study, HIP allogeneic islet cells transplanted without immunosuppression (IS) were not rejected, remained functional up to 12 weeks and achieved functional glucose-responsive insulin secretion. This provides a landmark proof of concept that immune evasive cells can be generated by gene modification and could overcome one of the central barriers to curative cell therapy for type 1 diabetes. By design, the functional mass of endocrine cells implanted was insufficient to reverse diabetes, and difficulties in upscaling the technique to a sufficient islet mass can be anticipated. It should be mentioned that, before going to the clinical setting, the authors had reported successful reversal of diabetes, using the same islet modification methodology, in a humanized mouse model transplanted with human HIP stem cell islets [24] and remarkably in an allogeneic non-human primate model [25].

## Stem Cells

The second landmark paper in the islet transplantation field reported the results of transplantation of stem cell-derived islets in 14 patients with type 1 diabetes [5]. This trial was led by the Vertex company and utilized a cell product baptized zimislecel, obtained by an *in vitro* differentiation protocol able to obtain large quantities of fully differentiated and glucose-responsive islets [26]. The trial was designed as phase 1-2 to determine safety and efficacy of the product, and the paper is an unplanned interim analysis of the first 12 patients to have received a full dose of the product and completed a 12-month follow-up. The primary efficacy endpoint was freedom from severe hypoglycemic events until day 365 after infusion, with a glycated hemoglobin level <7%. Remarkably, all patients met the primary endpoint and 10/12 patients were insulin-independent at 1 year. All patients who came off insulin did so after a period of several months, suggesting that further differentiation may have been taking place *in vivo* after transplantation [5]. These data demonstrate a formidable technological and clinical achievement and advance and provide the first evidence that stem cell-derived tissues can be successfully used as an organ replacement therapy. However, zimislecel administration was done similarly to islet transplantation and used an identical IS protocol; accordingly, the clinical outcomes were similar to those achieved with the landmark “Edmonton protocol” [27]. While zimislecel may become a solution in the USA, where allogeneic islet transplantation is essentially unavailable [28], several issues will have to be solved before it can become a real solution for bringing a cure to all type 1 diabetic patients [21]. The application of the immune evasiveness strategy described above [4] to stem cell-derived islets may become a solution to that end.

## Machine Perfusion

*Ex vivo* machine perfusion has rapidly developed in the last decade, driven by the ever-increasing gap between the numbers of donor organs and patients on the waiting list concomitantly with the increase in the proportion of extended criteria and marginal donors (older age, DCD, fatty livers, …). Hypothermic (HMP), hypothermic oxygenated and later, normothermic machine perfusion (NMP) have become a standard-of-care solution for the reconditioning of marginal organs [29, 30]. Machine perfusion also offers the possibility of assessing physical or biological parameters in the perfusate to provide information about organ quality, and thus transplantability [31].

The seminal multicenter European randomized controlled trial (RCT), comparing hypothermic kidney perfusion, using the Organ Recovery Systems machine, to cold storage enrolled >800 patients and its results were published already in 2009. The primary endpoint of the trial, i.e., occurrence of delayed graft function, was verified, thus demonstrating a significantly lower rate of delayed graft function in the perfusion group. Superior 1-year graft survival was also reported [32]. Better graft survival was still observed in a 3-year follow-up paper [33]. The article selected presents the long-awaited 10-year data [6]. Remarkably, the graft survival advantage was still present, and the long term observation revealed that this advantage was only conferred to expanded criteria donors [6]. This paper provides compelling evidence to recommend HMP for all kidneys procured from expanded criteria donors, notably DCDs.

Advances and utilization of machine perfusion technology have largely been driven by the kidney and liver transplantation fields. The amount of evidence gathered for thoracic organs has been less extensive and has mostly addressed lung transplantation [34, 35]. The rapid development of DCD heart transplantation in the past decade has provided an incentive for maximizing utilization of DCD hearts using machine perfusion technology. In contrast to kidneys and livers, NMP has been the preferred, and indeed sole, approach employed in this setting [36]. Unfortunately, and perhaps for understandable reasons, no prospective randomized trial is available to compare NMP to static cold storage (SCS) in DCD hearts.

The OCS NMP machine (Transmedics, Andover, MA, USA) is the only one approved for clinical use in the USA. It allows normothermic pulsatile perfusion during transportation of the heart from recovery to implantation. The selected paper is a Transmedics-led study combining data from the OPTN and OCS Heart Perfusion (OHP) registries and providing real-world data on the largest cohort (854 patients in 56 US centers) of NMP-preserved heart transplants ever reported [7]. The large numbers (>3,000 subjects, including OPTN data) and a rigorous methodology have allowed meaningful comparisons of DCD versus DBD cohorts, and NMP versus SCS strategies. The most striking result is the similar 1-year patient survival data in SCS-preserved DBD hearts and NMP-preserved DCD hearts (>90%), observed in spite of 2-3 times longer travelling distances and times. It is remarkable that >25% of hearts transplanted in the USA over the study period were preserved with NMP and <4% of procured NMP hearts were finally rejected [7]. In summary the study convincingly shows that NMP allows the recovery of hearts with longer shipping distances and from extended-criteria donors. This is likely to lead to the rapid normalization, in the USA and beyond, of NMP for the preservation of DCD and other marginal donor hearts.

## Transplant Oncology

Hepatocellular carcinoma (HCC) is the third most important indication for liver transplantation after alcohol-associated cirrhosis and metabolic dysfunction–associated steatohepatitis [37]. Recently, Immune checkpoint inhibitors (ICIs) have revolutionized the management of HCC and become standard-of-care as part of the treatment of advanced HCC [38]. This has allowed not only prolonged patient survival, but also tumour downstaging, bringing patients within Milan or other liver transplantability criteria. Unfortunately, the mechanism of action of ICIs, which essentially boosts the adaptive immune system against the tumour, has resulted in the early experience in a high risk of acute rejection episodes in ICI-treated patients receiving a liver transplant [38, 39]. A consensus has evolved to suggest that ICIs were a powerful neoadjuvant therapy for downstaging or bridging purposes, but that the optimal “washout” period (free interval between end of ICI treatment and liver transplantation) still had to be determined [40].

The selected paper is a multi-center retrospective study of 119 liver transplant patients treated with ICIs before liver transplantation in 29 transplant centers worldwide [8]. The authors analyzed the relationship between the washout period and several outcome measures, including occurrence and type of rejection, tumour recurrence and survival. They reported a 20% rejection rate, occurring early after transplantation, with a linear reverse relationship between rejection risk and washout period between 3 and 50 days. Beyond 50 days an increased rejection risk was no longer observed. Importantly, with a median follow-up of 18 months, patients with a longer washout period did not present a higher risk for HCC recurrence. There is a caveat with the fact that 9 different ICIs were used in these 119 patients, making it difficult to determine, due to low numbers, whether the 50-day cut-off was applicable equally to each of these molecules. Nonetheless, this is the first study to provide convincing evidence about the optimal washout period to observe and the uselessness of waiting times >50 days before performing liver transplantation in ICI-treated HCC patients.

The field of liver transplantation oncology is not limited to HCC, as unresectable colorectal liver metastases are increasingly becoming a valid indication for liver transplantation in selected cases. The ARTx-Onc study [41] provides a US perspective and favourable outcome results that slightly differ from those of the recent European Transmet RCT [42].

## Transplant Infectious Disease

BK polyomavirus nephropathy is a significant challenge for the transplant nephrologist, which can have a serious impact on kidney graft function and compromise graft survival in the most serious cases. The standard IS regimen associating tacrolimus, mycophenolate (MMF) and steroids is the primary risk factor for BK viral replication, with a high risk of developing BK nephropathy. The primary strategy in the management of BK virus infection is reduction of the IS at the risk of triggering graft rejection. Due to their antireplicative properties, mTOR inhibitors, well established IS drugs, have demonstrated potent *in vitro* antiviral activity, including against BK polyomavirus. Accordingly, RCTs exploring the efficacy of everolimus-based versus MMF-based IS regimens in kidney transplantation have also looked at the occurrence of BK infection and nephropathy. It was found that subjects receiving everolimus had experienced a lower rate of BK infection at 12 months, but none of these studies consistently monitored BK DNA levels [43, 44]. Therefore, the real role of a switch from MMF to everolimus remains undetermined and prompted the authors of this paper to launch a RCT comparing the efficacy of reducing MMF dosage versus switching from MMF to everolimus, alongside reduced CNIs, in kidney transplant recipients with BK DNAemia [9]. To their surprise, the authors found that BK virus clearance was achieved significantly more often and more rapidly in patients with MMF dosage reduction alone, compared to those switched to everolimus. Parameters of kidney function at the end of follow-up were identical in both groups [9]. These data challenge the notion that a switch to mTOR inhibitors will lead to faster clearance of BK viremia. On the contrary, it appears ineffective for managing BK DNAemia in kidney transplant recipients and cannot be recommended as a management strategy for BK replication control.

## Transplant Immunology

Minimization of donor-recipient HLA mismatches has been utilized as an allocation criterion for years as a strategy to avoid antibody and T cell–mediated rejection for most solid organ transplants. A role for the innate immune system in the pathogenesis of graft rejection has been unravelling in recent years [45, 46]. The main pathways thought to be involved are natural killer (NK) cell activation via missing self signals and monocyte activation through the signal regulatory protein α (SIRPα)-CD47 interaction. In the selected paper, the authors have studied the impact of mismatches in SIRPA, the gene encoding signal regulatory protein α (SIRPα), on the risk of renal allograft rejection [10]. They first determined that only 2 haplotype categories were present in >90% of the human population. In two independent cohorts of patients representing >700 subjects, they found that SIRPA mismatches were associated with an increased risk of renal allograft rejection, graft fibrosis and allograft survival. Differences in rejection-free and overall graft survivals were not only statistically significant, but also clinically relevant. For the most impactful mismatch type, acute rejection-free survival was 81% vs. 94%, and death-censored graft survival was 75% vs. 92%, both assessed at 7 years post-transplant [10]. In a murine model, they were able to show that SIRPα variants binding to CD47 elicit monocyte activation implicated in chronic allograft pathology. These results may impact both on matching strategies and on the therapeutic targeting of innate immunity and not only of T-cell activation. More specifically, with the simplicity of having only 2 haplotypes to consider and the high potential impact on kidney graft prognosis, the inclusion of SIRPA genotyping and matching could be an easy and efficient strategy to apply as a component of kidney graft allocation algorithms.

## Transplant Pathology

Since 1991, the Banff classification has been the international, consensus system that standardizes the biopsy-based diagnosis, classification and grading of kidney graft rejection. It provides a universal language for pathologists and clinicians that characterizes specific types of lesions (inflammation, tubulitis, arteritis, glomerulitis, …), and scores their severity to guide patient care and graft monitoring and to standardize reporting of outcomes [47].

With the reflection that the Banff classification is a dichotomic classification into discrete categories of a phenomenon that is a continuum in essence, the authors of this paper are proposing four continuous indices, easy to implement and interpret, for the global evaluation of kidney transplant histology [11]. Indices were developed from the analysis of nearly 20’000 biopsies from 10 centers worldwide. The formulas for these indices can be found in the article. The first 2 indices (AMR/MVI and TCMR/TI index) derived from routinely assessed histological lesion scores were designed to enable the quantification of the global spectrum of kidney transplant rejection, and the last 2 indices (Activity and Chronicity index) to replace the Banff diagnostic subcategories of rejection by more continuous activity and chronicity measures. The indices developed from a derivation cohort of >10’000 biopsies were verified in 2 separate validation cohorts of >5’000 and >1’000 biopsies respectively.

The four continuous measures of kidney transplant rejection capture much of the histological spectrum and severity of rejection while closely aligning with the current Banff classification. A significant interest of these indices is that it allows to better define, on a continuum basis, the nature, and thus the necessary intervention, for lesions characterized as “borderline” or “probable” rejection in the Banff system. Overall, this paper offers a refreshing replacement of rigid, threshold-based rejection categories with robust continuous indices capturing the true biological continuum of kidney allograft rejection. It provides a scalable framework that can directly refine clinical decision-making and trial endpoints in transplant nephrology. It has the potential of being a true game-changer in the classification of kidney allograft rejection in the very near future.

## Photopheresis

Chronic lung allograft dysfunction (CLAD) is an umbrella term coined to cover the different manifestations of irreversible lung allorejection, its most common form being bronchiolitis obliterans syndrome (BOS). Despite advances in diagnosis, little progress has been achieved in the prevention and treatment of this crippling condition, and CLAD remains the main cause for late pulmonary graft loss [48]. Recently, extracorporeal photopheresis (ECP) has emerged as a promising supportive treatment for the prevention and management of rejection in heart and lung transplants, with growing evidence supporting its use in kidney and liver transplants [49]. ECP is an extracorporeal therapy, combining leukapheresis with photoactivation. It consists in the incubation of the recipient’s mononuclear cells with a DNA-crosslinking molecule activated by UVA radiation, causing T-cell apoptosis, before reinfusing the cells into the patient. ECP was first developed for the treatment of T cell lymphomas and later used in other indications including graft-versus-host disease and organ cell-mediated rejection [49]. A recent multicenter study of lung transplant recipients started on ECP for a diagnosis of CLAD reported a response to treatment (stabilization or improvement) in about 50% of study subjects [50].

This article reports the outcomes of a RCT in which 62 patients with chronic obstructive pulmonary disease were randomized to receive ECP from day 2 to week 11 after lung transplant, in addition to conventional IS, or IS alone. The composite primary outcome measure was the occurrence of acute cellular rejection (ACR), cytomegalovirus infection, or CLAD within 2 years of transplantation [12]. ECP addition to IS resulted in a 3-fold decrease in meeting the primary endpoint (20% vs. 60%), mostly driven by a lower incidence of ACR or CLAD. Importantly, counting and phenotyping of circulating lymphocytes were similar in the treated and the control group, indicating that ECP had not led to overimmunosuppression, accounting for the absence of an increase in CMV infection.

This trial represents a potential game changer for the survival and quality of life of lung transplant recipients. It could redefine the standard IS protocol for lung transplantation if these results are confirmed in patients transplanted for other indications and in larger cohorts.

## Data Availability

The original contributions presented in the study are included in the article/Supplementary Material, further inquiries can be directed to the corresponding author.
