# Supplementary material for: The Top 12 Most Impactful Papers in Clinical Transplantation in 2025: TI Editors’ Choice
Source: Transpl Int. 2026 Feb 23;39:16247. doi: 10.3389/ti.2026.16247 (PMC12968042; doi:10.3389/ti.2026.16247)
Supplement: Supplementary file 1 [file Table1.docx]

Supplementary Table 1. Other impactful articles published in 2025 in clinical transplantation

| **Title** | **Topic** | **doi** |
| --- | --- | --- |
| Enzymatic conversion of blood group B kidney prevents hyperacute antibody-mediated injuries in ABO-incompatible transplantation | ABO-incompatibility | https://doi.org/10.1038/s41467-025-56563-w |
| Induction of immune education in type 1 diabetes through controlled allogeneic islet rejection at onset: a monocentric open-label pilot study | Immune tolerance | https://doi.org/10.1016/j.eclinm.2025.103685 |
| Randomized trial investigating the utility of a liver tissue transcriptional biomarker in identifying adult liver transplant recipients not requiring maintenance immunosuppression | Immune tolerance | https://doi.org/10.1016/j.ajt.2024.12.002 |
| Identification of indirect CD4+ T cell epitopes associated with transplant rejection provides a target for donor-specific tolerance induction | Immune tolerance | https://doi.org/10.1016/j.immuni.2025.01.008 |
| Exploring parvovirus B19 pathogenesis and therapy among kidney transplant recipients: case report and review of literature | Infectious diseases | https://doi.org/10.1016/S1473-3099(24)00560-7 |
| Low-Dose Valganciclovir for Primary Cytomegalovirus Prophylaxis After Heart Transplant: A 10-Year Experience | Infectious diseases | https://doi.org/10.1111/ctr.70408 |
| Safety and efficacy of immunoguided prophylaxis for cytomegalovirus disease in low-risk lung transplant recipients in Spain: a multicentre, open-label, randomised, phase 3, noninferiority trial | Infectious diseases | https://doi.org/10.1016/j.lanepe.2025.101268 |
| Cellular and humoral immunogenicity of respiratory syncytial virus vaccination in solid organ transplant recipients | Infectious diseases | https://doi.org/10.1016/j.ajt.2025.12.003 |
| Alternative Complement Pathway Inhibition with Iptacopan in IgA Nephropathy | Kidney transplantation | https://dx.doi.org/10.1056/NEJMoa2410316 |
| Survival Benefits of Deceased Donor Kidney Transplant vs Waitlisting | Kidney transplantation | https://doi.org/10.1001/jamainternmed.2025.5624 |
| A randomized controlled trial of intravenous immunoglobulin vs standard of care for the treatment of chronic active antibody-mediated rejection in kidney transplant recipients | Kidney transplantation | https://doi.org/10.1016/j.kint.2025.04.023 |
| Single-cell dissection of chronic lung allograft dysfunction reveals convergent and distinct fibrotic mechanism | Lung transplantation | https://doi.org/10.1172/jci.insight.197579 |
| Prognosis and Risks for Probable Chronic Lung Allograft Dysfunction: A Prospective Multicenter Study | Lung transplantation | https://doi.org/10.1164/rccm.202403-0568OC |
| Serum Ammonia Screening and Donor Mollicutes Detection for Hyperammonemia Syndrome Post-Lung Transplantation: A Prospective Observational Study | Lung transplantation | https://doi.org/10.1093/cid/ciaf078 |
| Long-term outcomes after hypothermic oxygenated machine perfusion and transplantation of 1,202 donor livers in a real- world setting (HOPE-REAL study) | Machine perfusion | https://dx.doi.org/10.1016/j.jhep.2024.06.035 |
| Outcomes of donation after circulatory death (DCD) and ex-vivo lung perfusion (EVLP) lung transplantation | Machine perfusion | https://dx.doi.org/10.1016/j.healun.2024.10.001 |
| Prolonged normothermic perfusion of the kidney prior to transplantation: a historically controlled, phase 1 cohort study | Machine perfusion | https://doi.org/10.1038/s41467-025-59829-5 |
| Successful AAV8 gene therapy on hepatic ex situ machine perfusion for mitochondrial neurogastrointestinal encephalomyopathy | Machine perfusion | https://doi.org/10.1016/j.jhep.2025.07.022 |
| Normothermic versus Hypothermic Machine Perfusion in Kidney Transplantation: a Randomized Controlled Trial | Machine perfusion | https://www.sciencedirect.com/science/article/pii/S1600613525031910 |
| GLP-1 receptor agonists in kidney transplant recipients with pre-existing diabetes: a retrospective cohort study | Metabolic disorders | https://dx.doi.org/10.1016/S2213-8587(24)00371-1 |
| Simultaneous liver transplant and sleeve gastrectomy provides durable weight loss, improves metabolic syndrome and reduces allograft steatosis | Metabolic disorders | https://doi.org/10.1016/j.jhep.2025.02.030 |
| Robot-assisted Kidney Transplantation: The 8-year European Experience | Robotic transplantation | https://doi.org/10.1016/j.eururo.2024.12.005 |
| Living-Donor Kidney Transplantation: Comparison of Robotic-Assisted Versus Conventional Open Technique | Robotic transplantation | https://doi.org/10.3389/ti.2025.14953 |
| Improved Short-Term Outcomes With Fully Robotic Recipient Adult Living Donor Liver Transplantation: A Comparative Study | Robotic transplantation | https://doi.org/10.1097/SLA.0000000000006807 |
| Effect of felzartamab on the molecular phenotype of antibody-mediated rejection in kidney transplant biopsies | Transplant immunology | https://doi.org/10.1038/s41591-025-03653-3 |
| Discovering molecular signatures in kidney transplant biopsies with borderline changes and isolated V-lesions: single-cell RNA-sequencing analysis of human blood and tissue Spatial transcriptomics | Transplant immunology | https://doi.org/10.1038/s41598-025-05191-x |
| Diagnostic significance of intragraft donor-specific anti-HLA antibodies in pulmonary antibody-mediated rejection | Transplant immunology | https://doi.org/10.1016/j.healun.2025.08.007 |
| Intention-to-treat outcomes of patients with hepatocellular carcinoma receiving immunotherapy before liver transplant: The multicenter VITALITY study | Transplant oncology | https://doi.org/10.1016/j.jhep.2024.09.003 |
| Downstaging of hepatocellular carcinoma before liver transplantation: Results from a national multicenter prospective cohort study | Transplant oncology | https://doi.org/10.1097/HEP.0000000000001231 |
| Liver Transplant for Upfront Unresectable Colorectal Metastases | Transplant oncology | https://doi.org/10.1001/jamasurg.2025.2879 |
| Physiology and immunology of pig-to-human decedent kidney xenotransplant | Xenotransplantation | https://doi.org/10.1038/s41586-025-09847-6 |
| Gene-modified pig-to-human liver xenotransplantation | Xenotransplantation | https://doi.org/10.1038/s41586-025-08799-1 |
| Pig-to-human lung xenotransplantation into a brain-dead recipient | Xenotransplantation | https://www.nature.com/articles/s41591-025-03861-x |
| Multi-omics analysis of a pig-to-human decedent kidney xenotransplant | Xenotransplantation | https://doi.org/10.1038/s41586-025-09846-7 |
